# Improved electroanalytical characteristics for flumetralin determination in the presence of surface active compound

**DOI:** 10.1007/s00706-017-1918-8

**Published:** 2017-02-08

**Authors:** Dariusz Guziejewski, Sylwia Smarzewska, Radovan Metelka, Agnieszka Nosal-Wiercińska, Witold Ciesielski

**Affiliations:** 1 Faculty of Chemistry, Department of Inorganic and Analytical Chemistry, University of Lodz, 92-236 Łódź, Poland; 2 Faculty of Chemistry, Department of Analytical Chemistry and Instrumental Analysis, Maria Curie-Skłodowska University, 20-031 Lublin, Poland; 3 Department of Analytical Chemistry, Faculty of Chemical Technology, University of Pardubice, Studentska 573, 53210 Pardubice, Czech Republic

**Keywords:** Square wave voltammetry, Herbicides, Plant growth regulator, Electrochemistry, Silver amalgam film electrode

## Abstract

**Abstract:**

The use of square wave voltammetry (SWV) and square wave adsorptive stripping voltammetry (SWAdSV) in conjunction with a cyclic renewable silver amalgam film electrode for the determination of flumetralin is presented. Poor separation of two overlapped reduction peaks is significantly improved when hexadecyltrimethylammonium bromide is used as a component of the supporting electrolyte solution (together with BR buffer pH 9.5). The SW technique parameters were investigated and found optimal as follows: frequency 50 Hz, amplitude 40 mV, and step potential 5 mV. Accumulation time and potential were studied to select the optimal conditions in adsorptive voltammetry. The analytical curve was linear for the flumetralin concentration range from 1.0 × 10^−6^ to 1.0 × 10^−5^ mol dm^−3^ and from 5.0 × 10^−9^ to 1.0 × 10^−7^ mol dm^−3^ for SWV and SWAdSV, respectively. Detection limit of 6.5 × 10^−10^ mol dm^−3^ was calculated for accumulation time 60 s at −0.2 V. The repeatability of the method was determined at a flumetralin concentration level equal to 5.0 × 10^−9^ mol dm^−3^ and expressed as %RSD = 5.0% (*n* = 6). The proposed method was applied and validated successfully by studying the recovery of herbicide content in spiked environmental samples.

**Graphical abstract:**

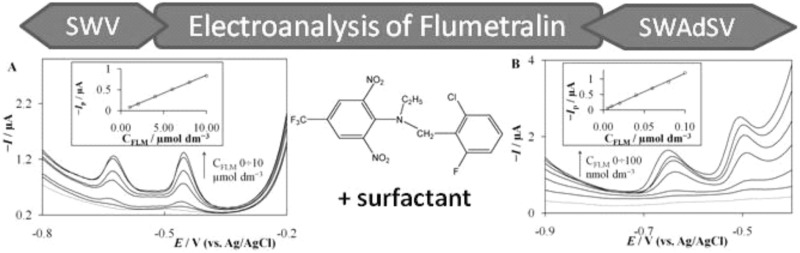

## Introduction

Serious hazard towards environment can be created when herbicides are used in agriculture. They can bring significant contamination problems because of being poisonous to aquatic life, plants, or animals. Moreover, it is important to determine the presence of those compounds in groundwater, surface water, and especially in drinking water due to possible risk to humans. Although enormous amount of chemical compounds applied every year possess an important role in crop protection, nevertheless, it can lead to undesirable contamination of the environment. That is why, there is a continuous necessity in elaboration of sensitive, inexpensive, and rapid methods for determination of agrochemicals [1, 2].

Flumetralin (FLM, CAS: 62924-70-3, Scheme 1) belongs to the class of dinitroanilines and is used mainly as plant growth regulator in tobacco crops to control axillary bud growth [3, 4]. It is hardly mobile to immobile (FAO Mobility Classes; *K*_oc_ range 24–183 k) in aerobic soil, and its average half-life amount remains present in the environment even after 3 years. Under anaerobic condition, FLM is decomposed with 42 days half-life. This compound is rather stable to hydrolysis. In turn, the potential of FLM bioaccumulation in aquatic organisms is still not well defined. Due to its high stability in soils, there is a potential for FLM to reach surface or groundwater water through flow off and/or runoff either as a solution or as settleable solids. This herbicide is classified as practically non-toxic to birds and honeybees on an acute oral exposure or acute contact basis. Although FLM demonstrates both acute and chronic toxicity to freshwater fish and freshwater/estuarine/marine invertebrates in laboratory studies, environmental exposure remains unknown due to lack of data. On the other hand, indirect effects to terrestrial or aquatic organisms cannot be excluded completely due to the possible effect on plants and, therefore, changes in food chain or habitat. According to EU legislation, default MRL of 0.01 and 0.05 mg kg^−1^ was set to fruits, vegetables, meat, and milk as well as coffee, tea, tobacco, and spices, respectively.

**Figure:**
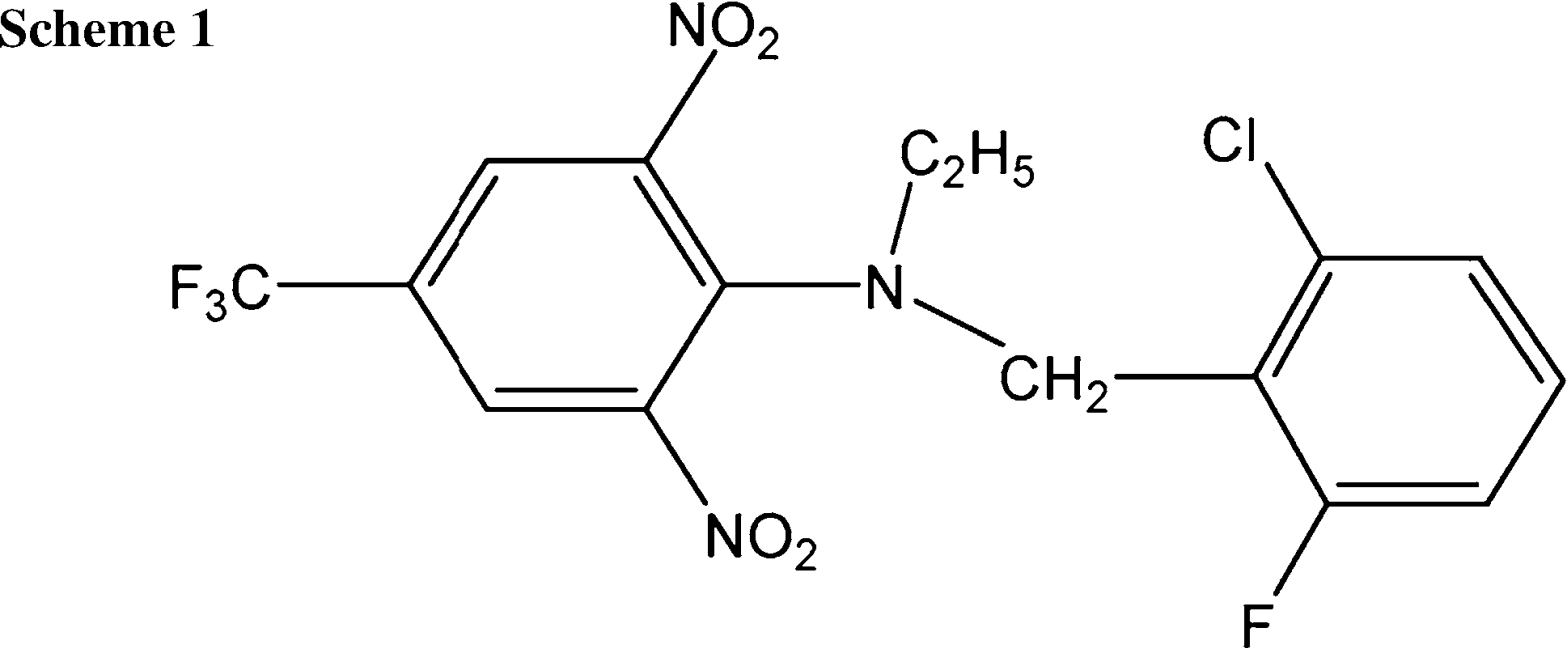

Up to date, only chromatographic methods have been used for the determination of trace amounts of FLM [5–13]. Most often, gas or liquid chromatography is combined with mass spectrometry [11–13] and preparation of the samples requires solid-phase extraction use. Unfortunately, the aforementioned methods require time and expenditures. Their suitability towards fast screening purposes is rather limited as several sample preparation steps are required (such as volume reduction, extraction, and clean-up procedures) to get compatibility with separation techniques. Furthermore, a large amount of organic solvents waste is generated, which results in further complication and raises of disbursements. On the other hand, the use of modern voltammetric techniques is rather fast, low-cost, and sensitive; therefore, it enables to use them in monitoring of electrochemically active compounds. Several different types of techniques [14–16] and working electrodes [17] have been proposed so far. Among them, the use of square wave voltammetry (SWV), especially in combination with accumulation, is often proposed [18–22].

Hanging mercury drop electrode as a working electrode would be obviously of first choice due to its extraordinary properties [23]. Nevertheless, modern electroanalytical methods suggest rather the use of modified solid [24–28] or amalgam electrodes [29, 30] as a substituent due to strict ecological and safety rules. Different types of amalgam or construction of electrodes have been proposed up to date. Among them, one can distinguish cyclic renewable silver amalgam film electrode [Hg(Ag)FE] from Polish group in Cracow [30–34], dental amalgam electrode from Norwegian group in Trondheim [35, 36], mercury meniscus modified silver solid amalgam electrode (m-AgSAE) from Czech group in Prague [37, 38], as well as amalgam modified screen-printed carbon electrodes [39, 40]. Surprisingly, up to date, no literature data can be found with respect to electrochemical determination of FLM.

Limitation in electrochemical analysis is often met due to overlapping of electron transfer signals. Insufficient peaks’ potential separation may result in difficulties in elaboration of electroanalytical methods. Nevertheless, if some surfactants are present in the supporting electrolyte solution, the effect on the electrochemical response can be quite useful. Surface active compound through their possible adsorption may influence the structure of the interface between the surface electrode and analyzed solution. It seems that some facilitation of other molecules’ adsorption and solubilization effects in the aggregates leads to the changes in redox potential, charge transfer, and diffusion coefficients of analytes. Therefore, the surface active compound presence may be useful in the improvement in electrochemical analysis selectivity and sensitivity. Several literature reports demonstrate possible detection and quantification of biologically active compound in the presence of interfering agents when appropriate surfactant is additionally present in the analyzed solution [41–44].

This paper presents electroanalytical measurements in the case of flumetralin herbicide. The studied compound reduction results in two overlapping signals. This problem was overcome in the presence of surface active compound. A validated procedure was successfully applied for FLM trace determination in several spiked environmental samples.

## Results and discussion

### Electrochemical behavior of flumetralin: influence of pH and SW parameters

FLM is an electroactive compound and square wave voltammograms recorded in BR pH 9.5 in its presence show two overlapped reduction signals, first close to −0.42 and second approximately at −0.52 V.

**Fig. 1:**
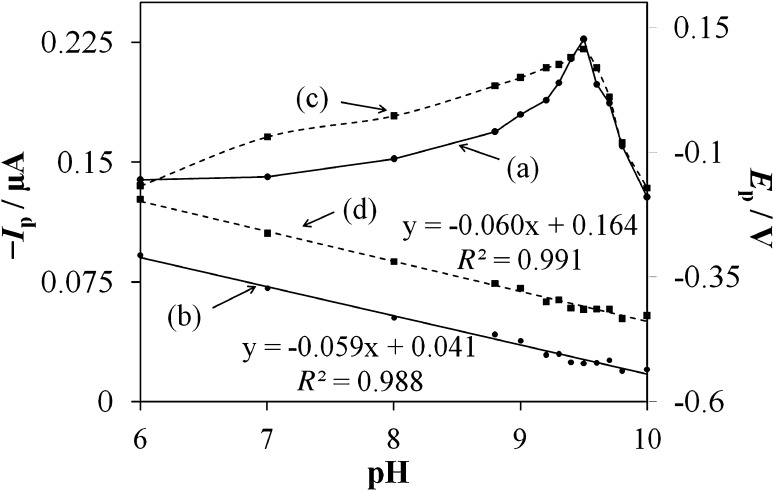
Effect of Britton–Robinson buffer pH on the SWV response of 5 × 10^−6^ mol dm^−3^ FLM. Plot shows peak current (*I*_p_) vs. pH and peak potential (*E*_*p*_) vs. pH dependencies of the signals at more (**a**, **b**) and less negative potential (**c**, **d**), respectively. Parameters of potential waveform: frequency 50 Hz, step potential 5 mV, amplitude 25 mV

The supporting electrolyte pH affected the electrochemical response of FLM and its influence was evaluated using the peak potential and current analysis (Fig. 1). The recorded signals of FLM were investigated in the pH range from 2.0 to 10.0 in 0.04 M BR buffer solution. FLM signals were apparent only in the 6.0–10.0 pH range. The observed peaks’ current was highly dependent on the supporting electrolyte pH. The maximum peak current was observed at pH 9.5 (Fig. 1, curve a). Above and below indicated pH both signals were significantly abated. Nevertheless, the peak potentials shifted towards more negative values with pH increment in the whole pH range, where signals were observed. The shift in cathodic peak potential with supporting electrolyte pH can be circumscribed with the following regressions: *E*_*p*1_ = 0.164–0.060 × pH, *R* ^2^ = 0.991 and *E*_*p*2_ = 0.041–0.059 × pH, and *R* ^2^ = 0.988 for signal observed at less and more negative potential, respectively. The slopes of the above equations are close to the theoretical value of 59 mV per pH unit and suggest involvement of protons in the FLM electroreduction most probably with equal number as of electrons. The BR buffer with pH 9.5 was selected as supporting electrolyte and applied in further experiments.

**Fig. 2:**
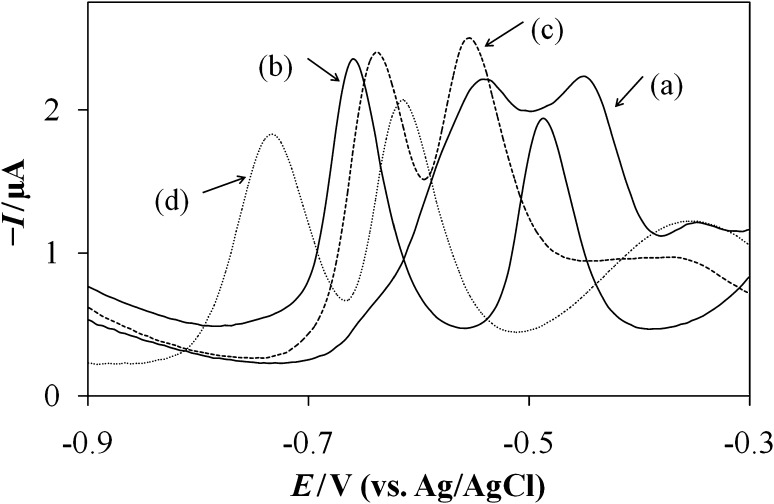
SW voltammograms recorded in Britton–Robinson buffer pH = 9.5 of 5 × 10^−6^ mol dm^−3^ FLM solution (**a**), also, in the presence of cetyltrimethylammonium bromide (**b**), sodium dodecyl sulfate (**c**), and Triton X-100 (**d**). Concentration of each surfactant was set to 1 × 10^−4^ mol dm^−3^. Parameters of potential waveform as in Fig. 1

As can be seen in Fig. 2a in pH 9.5 (and any other pH), FLM response has two vertices what could be related to reduction of two nitro groups in FLM structure. Those functional groups have only slightly different chemical surrounding causing very similar reduction potentials, and what is more probable the observed peaks are related to reduction of original and partly reduced molecule. The poor separation was overcome if surface active compound was present in the studied solution. The effect of surfactants on the voltammetric response of 5 × 10^−6^ mol dm^−3^ FLM at BR buffer pH 9.5 was investigated testing a cationic cetyltrimethylammonium bromide (CTAB, Fig. 2b), anionic sodium dodecyl sulfate (SDS, Fig. 2c), and neutral Triton-X 100 (Fig. 2d) surfactants. None of studied surface active compounds was electrochemically active in the studied potential range (data not shown). All the studied substances influence both FLM peak height and position (observed peaks’ potential separation for curves a–d was 90, 170, 85, and 120 mV, respectively). As can be noted the effect of an anionic and neutral surfactant is significant but not sufficient enough. However, in the presence of CTAB surfactant, the reduction signals were well shaped and markedly separated with additional shift towards more negative potential. The effect of the surfactant CTAB concentration from 1 × 10^−5^ to 1 × 10^−3^ mol dm^−3^ was studied with monitoring of the reduction response of 5 × 10^−6^ mol dm^−3^ FLM in BR buffer pH 9.5. The increasing CTAB concentration facilitated higher reduction peaks’ current until reaching a concentration of 5 × 10^−4^ mol dm^−3^ and it was followed with a decrease of the peak at a higher concentration. According to those findings analytical studies of FLM in BR supporting electrolyte pH 9.5 and presence of 5 × 10^−4^ mol dm^−3^ CTAB is highly recommended.

The optimization of square wave voltammetric parameters for FLM determination was an important stage in preparation of electroanalytical methodology. Significant influence of square wave voltammetric parameters on the FLM reduction signals was observed (data not shown). The amplitude (Δ*E*), the step potential (Δ*E*_s_), and the frequency (*f*) were studied within the variable ranges of 10–125 mV, 1–15 mV, and 8–250 Hz, respectively. The peaks’ current of FLM increased significantly with Δ*E* up to 40 mV, and remained constant up to 90 mV. Above this value minor, increase of the signal was noticed. However, higher values of amplitude caused severe broadening of the peaks, and therefore, value of 40 mV was chosen as an optimal amplitude based on the highest ratio between peak current and half peak width. The recorded FLM peaks’ shape and current was severely affected by applying a range of step potential values. The increase of the peaks’ height was linear in the aforementioned studied range. Nevertheless, the deterioration of signal shape was significant with higher step potential values. Therefore, primarily received signal shape was decisive for selection of step potential equal to 5 mV and this value was chosen for further analytical studies. In the range of studied square wave frequencies, a steady increase of peaks’ current was observed with *f* increment. Nevertheless, distinct signal shape deterioration was observed when frequency values higher than 50 Hz were applied. As the charging current increases also at higher frequencies, 50 Hz was selected for subsequent studies. The following values of square wave voltammetric parameters were found suitable for electroanalytical studies: frequency 50 Hz, amplitude 40 mV, and step potential 5 mV.

The cyclic renewable silver amalgam film electrode requires renewal and conditioning its surface as in the case of every solid working electrode. Therefore, the effect of the conditioning potential on the SWV signals of the fungicide determined the next optimization step. The conditioning potential (*E*_cond_) had a strong effect on the FLM peak current and peak potential. The effect of this parameter on the electrochemical behavior of 5 × 10^−6^ mol dm^−3^ FLM was studied for a conditioning time (*t*_cond_) 10 s in the potential range from −0.8 V to −2.0 V. It was found that in the investigated range, the potential influenced significantly the reduction peak current. At an *E*_cond_ more negative than −0.9 V, the FLM signals were descending. Conditioning at less negative potential caused the same effect. Selection of *t*_cond_ was conducted at *E*_cond_ = −0.9 V in the range from 0 to 30 s. Initial increase of peaks’ height was observed up to 6 s and further elongation of conditioning did not improve the characteristics of the FLM signals. Therefore, conditioning of the electrode in the case of FLM determination is recommended at −0.9 V for 6 s.

In the consecutive step, accumulation parameters were selected from the range 0–150 s and from 0.00 to −0.40 V for accumulation time (*t*_acc_) and potential (*E*_acc_), respectively. *E*_acc_ of −0.20 V was found to be optimal, since the peaks’ current achieved maximum height at this potential. Any deviation from this value caused significant drop of the recorded signals. Increase of the accumulation time resulted in consecutive increments of the peaks’ current up to 60 s and afterwards continuous decline. During both studies, the peaks’ potential shifted only slightly towards more negative potentials with analyzed parameter escalation. The most optimal accumulation potential −0.20 V and time 60 s were selected.

The electrochemical behaviour of FLM was, also, studied using cyclic voltammetry. The potential scan in previously chosen supporting electrolyte was started from 0.0 V towards the negative direction, and reversed at −1.0 V back to the starting potential. Flumetralin manifests two separated reduction peaks, related to irreversible process as there is no evidence of corresponding oxidation signals. The FLM peaks’ current was analyzed with scan rate range from 0.1 to 1 V s^−1^. Signals shifted towards more negative direction with the scan rate increment as expected from an irreversible reaction. Linear dependence of peak current and square root of scan rate were apparent (i.e., *I*_p_ = −1.84 × 10^−6^ *ν* ^1/2^ − 1.13 × 10^−7^, *R* ^2^ = 0.994 for the signal at more negative potential) indicating diffusion as a limiting process of electrode mechanism. This observation was confirmed by constructing the logarithmic dependence of peak intensity (A) versus the scan rate (V/s). The equation hold form log*I*_p_ = 0.52 × log*v* − 5.58 (*R* ^2^ = 0.990). The received slope was close to 0.5, which is attributed to diffusion-controlled processes [45].

The literature survey on other aromatic nitro compounds reduction [46, 47] as well as the presented above results suggested the following electrode mechanism. The recorded reduction signals could be connected with the reduction of two nitro groups present in the FLM structure. As a result of such electrode process, the NHOH groups were obtained as a product of a single 4e^−^, 4H^+^ irreversible step. The reduction of the second nitro-group proceeds on partly reduced molecule, so the recorded signals were observed at slightly different potential position. The consecutive peaks related to further reduction of hydroxylamine group to amino group is not visible in the studied potential range as it probably occurs at much more negative potential (at pH 6, it was observed at potential close to −0.8 V, data not shown). Moreover, those signals would be useless due to poor sensitivity towards FLM increasing concentration.

We would like to emphasize that both signals were analyzed with respect to the analytical purposes but due to increased stability and precision of the response under selected conditions and parameters, a signal at more negative potential value was chosen in further studies.

### Analytical application

**Fig. 3:**
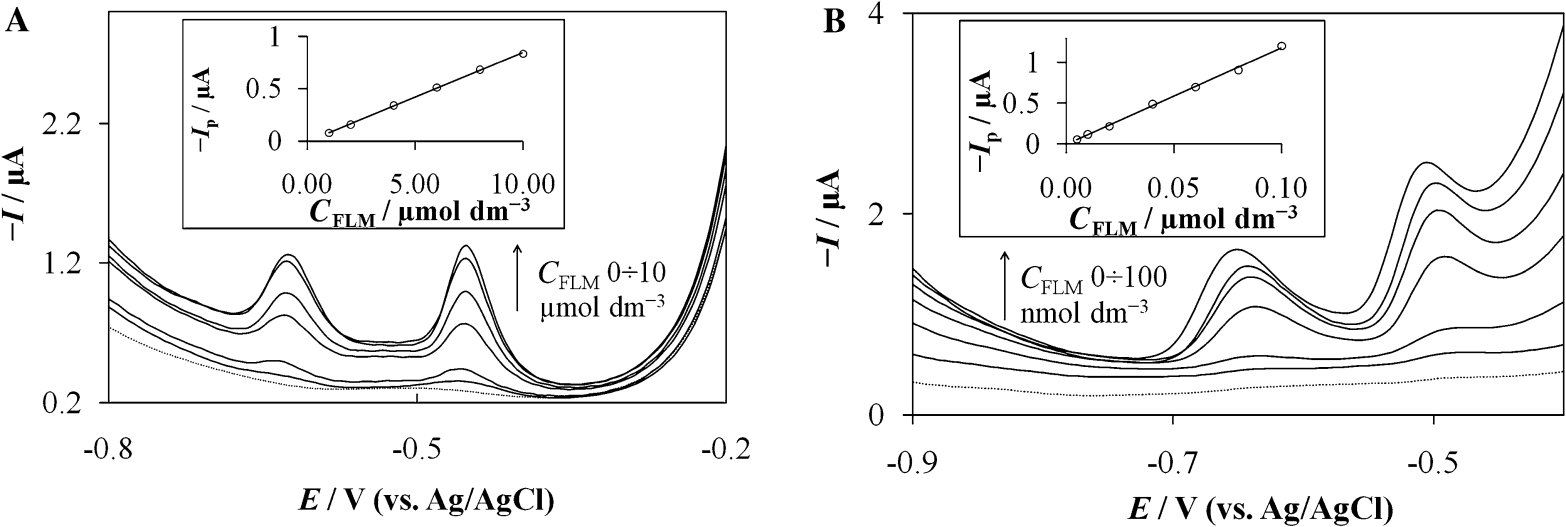
SWV (**a**) and SWAdSV (**b**) responses recorded in BR buffer pH 9.5 and 5 × 10^−4^ mol dm^−3^ CTAB with increasing FLM concentration from the bottom: **a** 0, 1.0, 2.0, 4.0, 6.0, 8.0, and 10 µmol dm^−3^; **b** 0, 5.0, 10, 40, 60, 80, and 100 nmol dm^−3^. The other experimental conditions were SW amplitude 40 mV, step potential 5 mV, frequency 50 Hz, accumulation time 60 s, and accumulation potential −0.2 V. *Inset* corresponding calibration line

Quantitative measurements of FLM were performed using square wave voltammetry (SWV) and square wave adsorptive stripping voltammetry (SWAdSV). The SWV and SWAdSV applicability for the determination of FLM was examined in the FLM concentration range 1 × 10^−6^–1 × 10^−5^ mol dm^−3^ and 5 × 10^−9^–1 × 10^−7^ mol dm^−3^, respectively (Fig. 3). Above those ranges, the linearity deviation was observed most probably due to saturation of the electrode surface with both surfactant and analyte. The calibration curves for both techniques were constructed with plotting the peak height against FLM concentration. The summary of calibration plot characteristics is placed in Table 1.

**Table 1:** Quantitative determination of FLM in BR buffer, pH = 9.5 with SWV and SWAdSV, and respective basic statistic data of the regression lines

	SWV	SWAdSV
Linear concentration range/mol dm^−3^	1.0 × 10^−6^–1.0 × 10^−5^	5.0 × 10^−9^–1.0 × 10^−7^
Slope of calibration graph/A dm^3^ mol^−1^	0.85 ± 0.01	11.85 ± 0.27
Intercept/A	(5.0 ± 0.3) **×** 10^−9^	(4.0 ± 0.2) **×** 10^−9^
Correlation coefficient, *R* ^2^	0.997	0.999
Number of measurements	6	6
LOD/mol dm^−3^	1.2 **×** 10^−7^	6.5 **×** 10^−10^
LOQ/mol dm^−3^	4.1 **×** 10^−7^	2.2 **×** 10^−9^

Both detection (LOD) and quantification limits (LOQ) of FLM determination were calculated using the equations: LOD/LOQ = *k*SD/*b* (*k* = 3 for LOD, *k* = 10 for LOQ, *b*, slope of the calibration curve) [48] for both techniques. The intra-day repeatability of the developed methods was tested for each studied FLM concentration from six replicate measurements. Correctness of the methods was checked with precision (%RSD) and recovery studies and, also, calculated at different flumetralin concentrations from the linear range response (Table 2).

**Table 2:** Recovery and precision of the FLM peak currents at various FLM concentrations, *n* = 6

Concentration /µmol dm^−3^		%RSD/%	Recovery/%
Given	Found		
SWV			
1.00	0.99	8.6	98.6
2.00	1.91	7.9	95.6
4.00	4.05	8.9	101
6.00	6.07	6.9	101
8.00	8.08	6.3	101
10.0	9.88	9.0	98.8
Concentration /µmol dm^−3^			
SWAdSV			
5.0	5.3	5.0	105
10	11	4.8	104
20	19	2.3	94.1
40	42	3.3	105
60	60	3.5	99.4
80	77	1.9	96.5
100	100	3.7	102

### Analysis of flumetralin in spiked environmental samples

**Fig. 4:**
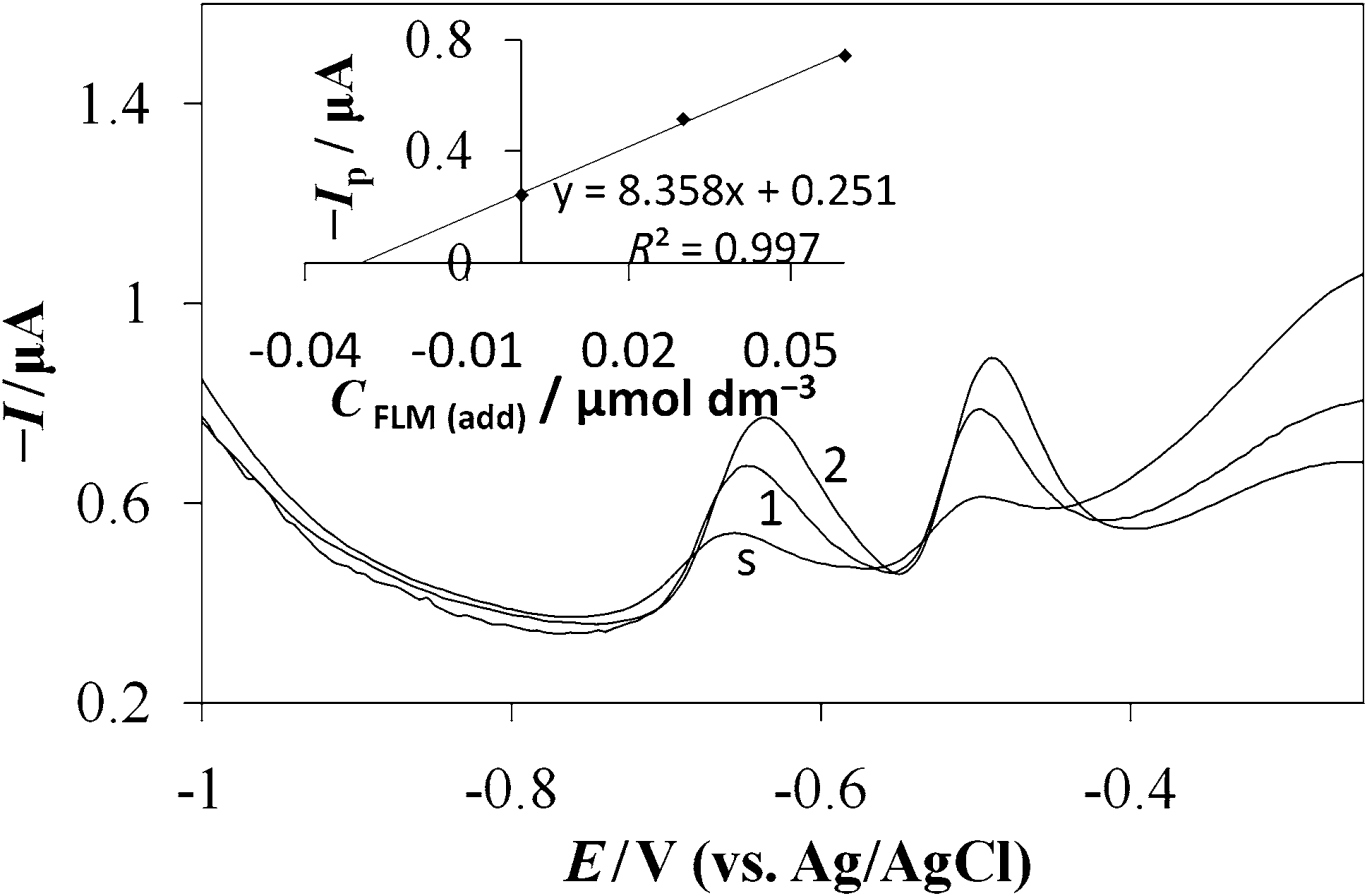
SWAdS voltammograms of FLM determination in spiked Elbe river water samples using the standard addition method (s, sample;* 1*,* 2*, standard additions). Experimental conditions are the same as in Fig. 3. *Inset* corresponding calibration curve

The reliability of the proposed SWV and SWAdSV method for the determination of FLM was investigated by assaying this compound in water samples. A series of spiked samples were used to investigate further the accuracy. Six replicate experiments were performed along with the standard addition method to determine FLM in spiked environmental samples. The standard addition method was used as a method of evaluation FLM content. The tap and river water as well as apple juice samples were spiked with different amounts of FLM with known concentrations (Table 3). It is worth noting that no matrix effects, nor signal shifting and nor deterioration of the peaks’ shape was observed (Fig. 4). There was no need for any evaporation, precipitation, or extraction steps prior to the pesticide assay. The applicability of the developed method for FLM determination was tested successfully by the standard addition method. Additional peaks were not observed within the examined potential window in the studied samples. FLM recovery results calculated from the linear regression equations are given in Table 3. The received results imply that the evaluated method is accurate, selective, and precise sufficiently enough to be introduced in routine analysis.

**Table 3:** Results of FLM determination in spiked samples with SWAdSV, *n* = 6

Sample	Concentration/mol dm^−3^		%RSD/%	Recovery/%
	Given	Found		
Tap water	3.0 × 10^−8^	(3.1 ± 0.1) × 10^−8^	2.2	102
River (Elbe)		(3.1 ± 0.1) × 10^−8^	2.6	102
River (Moszczenica)		(3.0 ± 0.1) × 10^−8^	2.1	101
Apple juice	4.0 × 10^−8^	(4.1 ± 0.1) × 10^−8^	1.4	102

### Interferences

Some of heavy metal ions and other agrochemicals were selected for the selectivity studies. The interferent concentration was increased in the range from 2.5 × 10^−8^ up to 5.0 × 10^−6^ mol dm^−3^. The recorded voltammograms were analyzed along with the result received only in the presence of FLM solution at the concentration 1.0 × 10^−7^ mol dm^−3^. The presence of cobalt and zinc ions interfered with the FLM voltammetric response in all studied concentration range, while lead and copper ions did not influence FLM signal at all. A constant decrease of FLM signal in the presence of increasing concentration of nickel ions suggests formation of a complex compound. In the case of any studied heavy metal ion, no additional signal was observed close to FLM signals. Blasticidine S and dinotefuran had no interference action in the studied concentration range. Aclonifen and nitrothal-isopropyl precluded FLM determination only at the concentration higher than 5.0 × 10^−7^ mol dm^−3^. Their additional signal was observed close to the signal at more and less negative signal from FLM, respectively. Nevertheless, one of the FLM signal was not affected at the same time. Methidathion interfered at concentration higher than 5.0 × 10^−6^ mol dm^−3^ (with only signal deterioration and not presence of additional signal), while thiophanate methyl caused significant distortion of recorded FLM voltammetric signal in all studied concentration ranges due to the presence of its relatively large, overimposing signal at approximately −0.68 V.

## Conclusion

The described above data clearly demonstrate the possible use of the cyclic renewable silver amalgam film electrode for square wave voltammetric and square wave adsorptive stripping voltammetric determination of flumetralin. The proposed methodology is fast, of high precision and accuracy, therefore, can be used for FLM quantification in samples with no matrix effects on the measurable response. The data acquired using the optimized experimental conditions and voltammetric parameters confirm the possible practical application and correctness of the proposed methodology. The delivered procedure ensured a new instrument for quantification of FLM in contaminated samples. When comparing to chromatographic methods, our electrochemical method seems to be of similar sensitivity. Only some of chromatographic methods combined with mass spectrometry detection are more sensitive [11, 12], while the others are less [6, 8, 9, 13]. Quantification limit of our method is below the proposed MRL within European Union. The newly developed procedure allows accurate detection of flumetralin and seems to be simple, fast, and highly sensitive. The capability to determine the plant growth regulator content directly from the matrix medium or natural samples without any laborious pretreatment which are usually time-consuming and environmentally unfriendly is one of the main advantages of the method. The presented voltammetric methods of FLM determination can be distinguished as sensitive and effective techniques and an alternative to expensive chromatographic methods for routine analysis of environmental samples or at least simple prescreening detection. The cyclic renewable silver amalgam film electrode can be directly used in field analysis due to its easy film regeneration and mechanical stability in contrast to the classic mercury electrodes.

## Experimental

### General voltammetric procedure, instrumentation, and software

A multichannel mAutolab with M101 potentiostat and the Nova 1.10.3 software (both Metrohm Autolab, Netherlands) were used for the measurements. The standard three-electrode cell consisted of a renewable silver amalgam film electrode (Hg(Ag)FE, refreshed, and conditioned before any measurement, surface area 12 mm^2^) as the working electrode, a reference electrode Ag/AgCl/KCl (3 mol dm^−3^), and an auxiliary electrode in a form of platinum wire. pH measurements were performed with a laboratory pH-meter CD-315 M (Elmetron, Poland). All experiments were performed at ambient temperature.

Electrochemical measurements with FLM were performed using square wave voltammetry (SWV) and square wave adsorptive stripping voltammetry (SWAdSV). Renewable silver amalgam film electrode (Hg(Ag)FE) was refreshed mechanically before any measurement. Typical measurement was performed in Britton–Robinson (BR) buffer pH 9.5 under the following conditions: conditioning potential −0.9 V for 6 s, accumulation (if applicable) at −0.2 V for 60 s, scan of the potential using square wave mode in a negative direction from 0 up to −1.5 V with step potential 5 mV, amplitude 40 mV, and frequency 50 Hz. All measurements were carried out in solutions deaerated with argon (10 min for the first time, 60 s for subsequent recording).

### Solutions and materials

No further purification was applied to any of the purchased analytical grade reagents. Standard stock solution of 0.001 mol dm^−3^ FLM (Dr Ehrenstorfer Gmbh) was obtained by dissolution and sonication of an appropriate amount in ethanol. Diluted solutions of FLM were prepared daily from the standard stock solution. BR buffer (0.04 mol dm^−3^) was prepared by mixing the corresponding amounts of phosphoric, acetic, and boric acid (POCh) with sodium hydroxide (Sigma-Aldrich). Cetyltrimethylammonium bromide (CTAB), sodium dodecyl sulfate (SDS), and Triton-X-100 were purchased from Sigma-Aldrich. All solutions were prepared with double-distilled water.

### Analysis of environmental samples

Spiked samples solutions were prepared after dissolving appropriate amount of FLM standard solution in a 50 cm^3^ flask and filled up to volume with tap (local delivery), river [Elbe (Pardubice), Moszczenica (Strykow-Lodz)] water, and apple juice. During voltammetric determination, the supporting electrolyte contained 2 cm^3^ of spiked (tap/river) water/juice solution, 8 cm^3^ of BR buffer with pH 9.5, and 50 mm^3^ of CTAB standard solution. The FLM concentration in spiked samples was analyzed using the standard addition method. Each addition contained 0.3 nmol of flumetralin in the case of water samples and 0.2 nmol of FLM when apple juice was analyzed. Voltammograms were recorded after each addition. Recoveries were calculated after six runs.
